# Aplastic Anemia With Thrombosis Following the Administration of Immunosuppressant and Thrombopoietin Receptor Agonist (TPO-RA)

**DOI:** 10.7759/cureus.61135

**Published:** 2024-05-26

**Authors:** Tadafumi Iino, Masako Yokoo, Sho Okamoto, Seiji Kondo

**Affiliations:** 1 Department of Blood Transfusion/Hematology, Saga-Ken Medical Centre Koseikan, Saga, JPN; 2 Department of Hematology, Saga-Ken Medical Centre Koseikan, Saga, JPN; 3 Department of Internal Medicine, Faculty of Medicine, Saga University, Saga, JPN

**Keywords:** thrombopoietin receptor agonist, thrombopoietin, thrombosis, eltrombopag, aplastic anemia

## Abstract

Thrombopoietin receptor agonist (TPO-RA) is effective for aplastic anemia (AA) and idiopathic thrombocytopenic purpura (ITP). However, the risk of thrombosis during ITP treatment with TPO-RA is higher than without TPO-RA. It is unclear whether TPO-RA increases the risk of thrombosis in patients with AA. We report a case of a 66-year-old female with severe AA having paroxysmal nocturnal hemoglobinuria (PNH) clones in the peripheral blood who developed ischemic colitis after three days of starting eltrombopag. Contrast-enhanced computed tomography showed ischemic colitis and contrast enhancement defect in the left atrial appendage, which indicated a thrombus in the heart. Stopping eltrombopag and providing supportive care improved her symptoms, and her blood cell counts gradually increased. Thrombosis should be considered when TPO-RA is administered during the immunosuppressive treatment of AA.

## Introduction

In treating aplastic anemia (AA), thrombopoietin receptor agonists (TPO-RAs), such as eltrombopag and romiplostim, have improved response rates when combined with immunosuppressive therapy [1,2]. Eltrombopag is a small oral molecule that binds to the TPO receptor and promotes hematopoiesis and platelet production. TPO receptor is expressed in hematopoietic stem cells, precursor cells of the megakaryocytic lineage, megakaryocytes, and mature platelets [3]. TPO-RA is not directly associated with exacerbating platelet functions such as platelet aggregation. However, clinically, especially in treating immune thrombocytopenia (ITP), TPO-RA administration increases the risk of arterial and venous thrombotic events during the platelet recovery period and periods of low platelet count, according to meta-analysis [4]. Cases of thrombosis and ischemic colitis during treatment with eltrombopag for AA are rare, but they are considered significant complications that warrant attention and reporting.

## Case presentation

A 66-year-old female had a complaint of fatigue around June 2019. She was referred to our hospital in August 2019 after her family doctor found that her white blood cell count was 2600/µL, her hemoglobin level was 8.6 g/dL, and her platelet count was 36000/µL. She had a history of hypertension, chronic heart failure, and atrial fibrillation and was taking antihypertensive medications and warfarin. She was admitted to our hospital in September 2019 for a further evaluation of her pancytopenia. On admission, she was clearly conscious, with a temperature of 36.4°C, a pulse of 90 beats/min, a blood pressure of 116/76 mmHg, and a percutaneous blood oxygen saturation of 98% (room air). She had conjunctival pallor and no abdominal tenderness. Blood tests showed pancytopenia and coagulation system tests showed prolonged prothrombin time due to warfarin's anticoagulation for atrial fibrillation (Tables 1-4). We adjusted the warfarin dosage so that the prothrombin time-international normalized ratio (PT-INR) remained in the therapeutic range, i.e., around 2 (1.6-2.6), before the onset of ischemic enteritis after hospitalization.

**Table 1:** Laboratory findings on admission (complete blood count).

Parameters	Results	Normal range
White blood cells	2.4×10^3^/μL	3.3-8.8×10^3^/μL
Segmented neutrophils	40.0%	38-74%
Lymphocytes	53.0%	16.5-49.5%
Monocytes	4.0%	2.0-10.0%
Eosinophils	1.0%	0.0-8.5%
Basophils	1.0%	0.0-2.5%
Atypical lymphocytes	1.0%	0.00%
Red blood cells	197.0×10^4^/μL	386-492×10^4^/μL
Reticulocytes	1.2%	0.8-2.2%
Hemoglobin	7.4 g/dL	11.6-14.8 g/dL
Hematocrit	22.0%	35.1-44.4%
Platelets	1.8×10^4^/μL	15.8-34.8×10^4^/μL

**Table 2:** Laboratory findings on admission (blood chemistry).

Parameters	Results	Normal range
Total protein	6.4 g/dL	6.6-8.1 g/dL
Albumin	4 g/dL	4.1-5.1 g/dL
Total bilirubin	0.7 mg/dL	0.4-1.5 mg/dL
Aspartate aminotransferase	24 IU/L	13-30 IU/L
Alanine aminotransferase	17 IU/L	7-23 IU/L
Lactate dehydrogenase	298 IU/L	124-222 IU/L
Alkaline phosphatase	195 IU/L	106-322 IU/L
Blood urea nitrogen	22.2 mg/dL	8.0-22.0 mg/dL
Creatinine	0.61 mg/dL	0.46-0.79 mg/dL
Sodium	145 mEq/L	138-145 mEq/L
Potassium	4 mEq/L	3.6-4.8 mEq/L
Chlorine	112 mEq/L	101-108 mEq/L
C-reactive protein	0.03 mg/dL	0.00-0.14 mg/dL

**Table 3:** Laboratory findings on admission (coagulation).

Parameters	Results	Normal range
Prothrombin time	16.1 s	10-13 s
Prothrombin time-international normalized ratio	1.44	0.9-1.1
Activated partial thromboplastin time	28.6 s	25-40 s
Fibrinogen	456 mg/dL	200-400 mg/dL
D-dimer	0.5 μg/mL	0.0-1.0 μg/mL

**Table 4:** Laboratory findings on admission (bone marrow aspiration).

Parameters	Results	Normal range
Nucleated cell count	1.5×10^4^/μL	10-25×10^4^/μL
Megakaryocytes	6.0/μL	50-150/μL
Erythroid cells	1.2%	15.0-36.2%
Myeloid cells	16.8%	34.7-78.8%
Lymphocytes	76.0%	8.6-23.8%
Monocytes	4.0%	0.0-0.6%
Plasma cells	1.4%	0.0-3.5%
Myeloblasts	0.0%	0.1-0.7%

**Figure 1:**
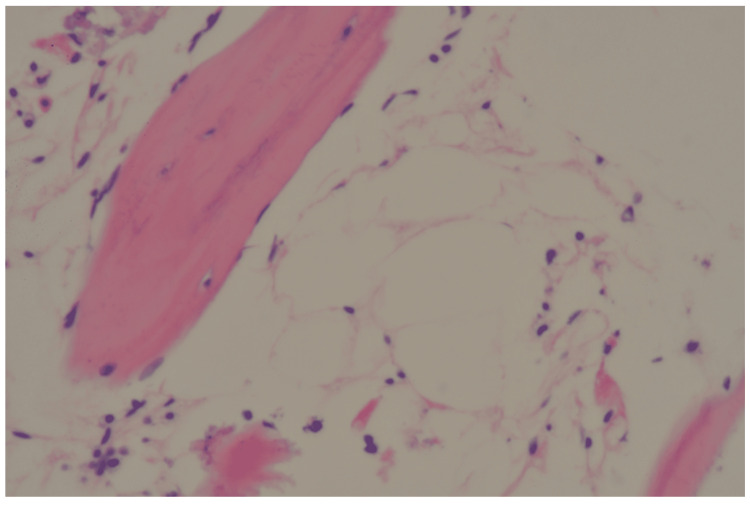
Bone marrow pathology. Hypoplastic marrow with some lymphocytes was observed. There were few megakaryocytes in the microscope field of vision.

**Figure 2:**
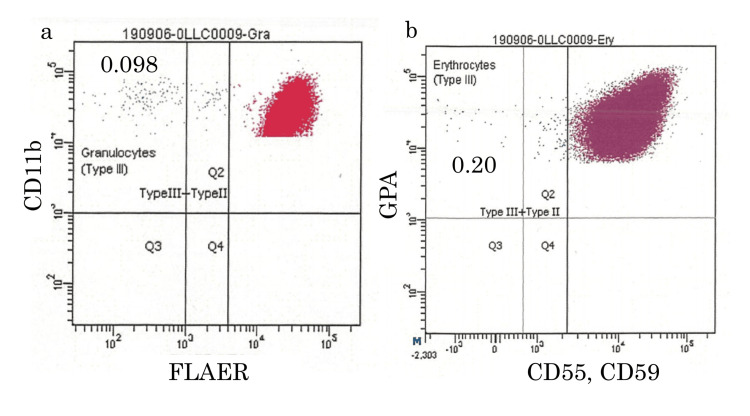
PNH clones in peripheral blood. The images show (a) PNH granulocytes (CD11b+ FLAER-) and (b) PNH erythrocytes (CD55-CD59-GPA+) FLAER: fluorescent-labeled inactive toxin aerolysin; GPA: glycophorin A; PNH: paroxysmal nocturnal hemoglobinuria

Based on her peripheral blood data and MRI findings of the bone marrow in her spine, which showed a high degree of fatty marrow with the high-intensity signal on TI-weighted images and a low-intensity signal on short tau inversion recovery (STIR) images, she was suspected of having AA. A bone marrow biopsy image showed hypoplastic bone marrow (Figure 1). Electrocardiography showed atrial fibrillation, but transthoracic echocardiography showed no evidence of intracardiac thrombosis. High-sensitivity paroxysmal nocturnal hemoglobinuria (PNH) blood cell analysis using flow cytometry showed 0.098% of type III PNH neutrophils, which were negative for fluorescent-labeled inactive toxin aerolysin (FLAEAR), and 0.20% of type III PNH erythrocytes were negative for CD55 and CD59 (Figures 2a, 2b).

**Figure 3:**
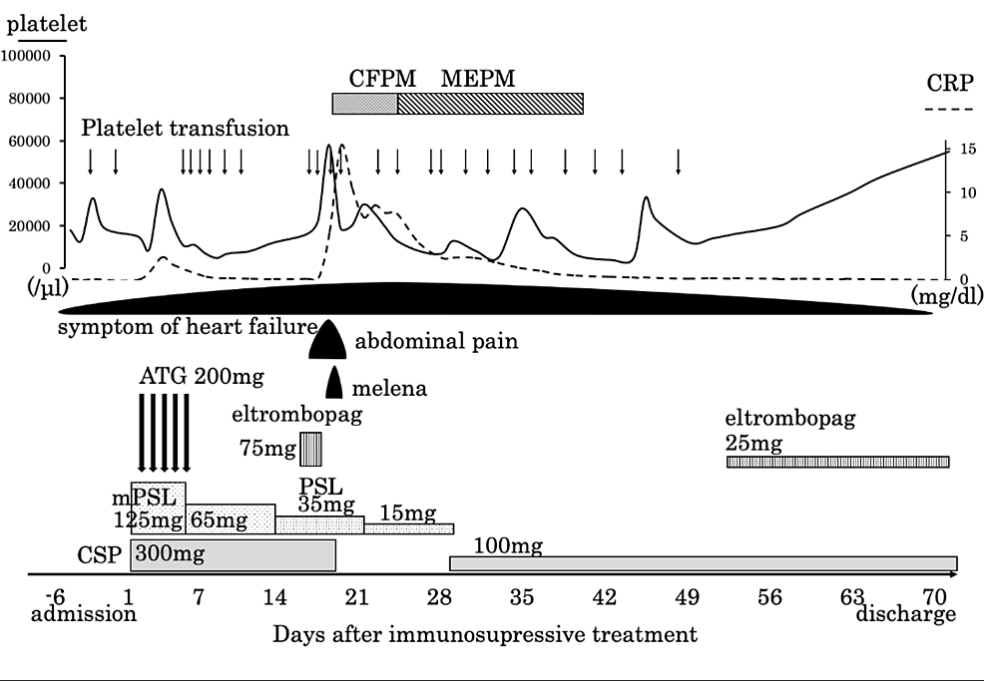
Clinical course since the start of treatment. CFPM: cefepime; MEPM: meropenem; PSL: prednisolone; CRP: C-reactive protein; ATG: antithymocyte globulin

We diagnosed her with severe aplastic anemia based on peripheral blood data and bone marrow pathology using Camitta criteria [4]. Figure 3 shows her clinical course since the start of treatment. Aplastic anemia was treated with antithymocyte immunoglobulin (ATG), immunosuppressive therapy with cyclosporine (CSP), and steroids.

**Figure 4:**
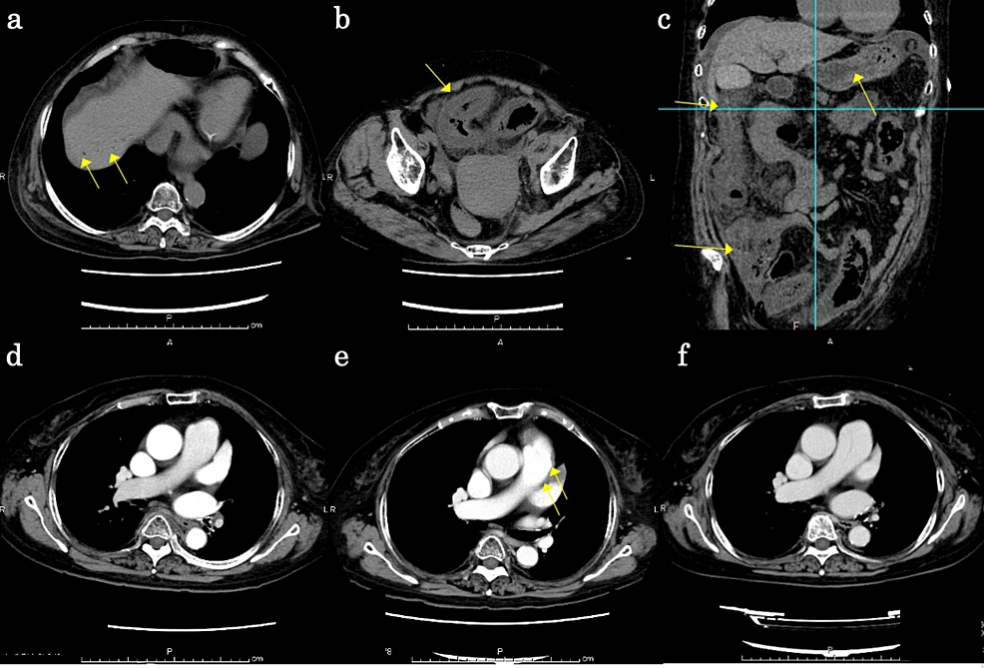
Abdominal and chest contrast-enhanced CT. CT scan showed ischemic colitis of the colon, free air in the liver portal vein (a), thickening of the gut wall in the transverse plane (b), and thickening of the gut wall in the coronal plane (c). CT scan of the chest showed complete enhancement of the left atrial appendage 10 days before the day of colitis (d), contrast enhancement defect on the day of colitis (e), and recovered enhancement of the left atrial appendage 10 months after the day of colitis (f).

We administered 200 mg of thymoglobulin as ATG and 125 mg of methylprednisolone intravenously for five days and initiated oral administration of 300 mg (5 mg/kg/day) of CSP. Then methylprednisolone was tapered off. Fever, allergic symptoms, and serum sickness-like symptoms associated with the use of rabbit-derived ATG were not seen due to the concomitant use of high-dose steroids. On the 18th day after the start of immunosuppressive therapy, the patient complained of constipation and abdominal pain, and a contrast-enhanced CT scan was performed, but there were no significant findings. On the 15th day of immunosuppressive therapy, eltrombopag 75 mg was started orally, and on the third day of eltrombopag (18th day of immunosuppressive therapy), abdominal distention and abdominal pain re-appeared, and there was marked increase in intestinal gas and melena appeared. She developed hemodynamic instability, and her systolic blood pressure dropped to 70 mmHg. We transfused her with red blood cells and platelets and managed her circulatory failure. Her white blood cell count was 2600/µL, her hemoglobin level was 6.1 g/dL, and her platelet count was 13000/µL when her condition suddenly changed. Abdominal and chest contrast-enhanced CT showed edema of the colon and intestinal tract, opacity of the surrounding fatty tissue, and air in the portal vein, which suggested ischemic enteritis, and filling defect image in the left auricle, which appeared to be a thrombus (Figures 4a-4f). During the thrombotic event, the D-dimer increased up to 7.9 μg/mL (normal range: 0.0-1.0 μg/mL), which indicates that thrombosis had occurred, and it took two months for the D-dimer to return to the normal range. The PT-INR at the time of the ischemic enteritis was 2.56 and was controlled within the therapeutic range. In this case, liver and renal function were routinely monitored with blood tests before and after the thrombotic event. However, no hepatic or renal dysfunction due to TPO-RA administration was observed during the observation period.

The platelet count remained around 10000/µL during the clinical course, and we transfused platelets when the platelet count fell below 10000/µL. After the onset of gastrointestinal symptoms, she was made nil per os, given broad-spectrum antibiotics, and discontinued eltrombopag, CSP, and warfarin. Her general condition gradually improved, and she resumed eating three days after the onset of ischemic enteritis. CSP was resumed at 100 mg/day on day 29 after the start of immunosuppressive therapy, and eltrombopag was resumed at 25 mg/day on day 51. She did not receive any transfusion after platelet transfusion on day 50 after the start of immunosuppressive therapy, and she resumed taking warfarin for atrial fibrillation on day 40 of immunosuppressive therapy. She was discharged from the hospital 68 days after the start of immunosuppressive therapy and is still being followed in the outpatient. There was no evidence of thrombocytopenia or new thrombus, and a CT scan 10 months after the start of immunosuppressive therapy showed that the thrombus in the heart had been obscured.

## Discussion

Aplastic anemia is a rare hematologic disease and is a hematopoietic disorder caused by immunologic mechanisms to hematopoietic stem cells. Clinically, it is characterized by pancytopenia, with associated fatigue, bleeding, and infection as the main symptoms. The severity of AA should be assessed according to the Camitta criteria based on marrow cellularity, neutrophils count, platelet count, and reticulocyte count. We diagnosed this patient with severe aplastic anemia based on marrow cellularity <25%, platelet count <20000/µL, and reticulocyte count <60000/µL. The recommended primary treatment for severe aplastic anemia is ATG+ CSP+/- eltrombopag in patients over 40 years [2,5]. Complications of this treatment, particularly associated with ATG, include allergic reactions due to heterologous proteins, transient cytopenia associated with antibody reactions, infections due to immunodeficiency, and secondary clonal abnormalities. In the present case, transient thrombocytopenia and neutropenia developed after ATG administration, resulting in increased platelet transfusions and filgrastim administration as granulocyte colony-stimulating factor (G-CSF) increased. High-dose steroids in combination with ATG and G-CSF may have contributed to the development of thrombosis in this patient. Eltrombopag is an oral TPO-RA associated with thrombosis and hepatic and renal dysfunction, stimulating the megakaryocyte's hematopoiesis. Eltrombopag has been shown to effectively increase platelets in AA and ITP and myelodysplastic syndrome (MDS) by stimulating megakaryopoiesis. Endogenous TPO binds to the extracellular domain of TPO receptors. In contrast, eltrombopag binds to the transmembrane domain of TPO receptors on blood cells and stimulates platelet production via the Janus kinase/signal transducers and activators of transcription (JAK/STAT) pathway [3,6]. Although eltrombopag is not directly involved in platelet activation in vitro and in vivo, it has been reported to increase the risk of thrombosis in clinical practice, especially in treating ITP [6]. A meta-analysis showed that thrombosis occurred in 3.69% of patients treated with TPO-RA, significantly more than in controls not treated with TPO-RA. The reason why TPO-RA causes thrombosis is unknown. The mechanism of TPO-RA-induced thrombosis is that TPO-RA promotes platelet aggregation by increasing p-selectin, an adhesion molecule on the platelet surface [7]. A search of case reports of thrombosis associated with AA, not limited to TPO-RA, revealed 12 cases, including the present case (Table 5).

**Table 5:** Patient characteristics of reported cases of aplastic anemia (AA) with thrombosis.

Estimated cause of thrombosis	Vessel/organ of thrombosis	Platelet count (/μL)	Treatment for thrombosis	Outcome	References
NA (not available)	Splenic artery, pulmonary vein	Absent	None	Death	^[8]^
Metenolone enanthate	Coronary artery	40000	None	Improved	^[9]^
Oxymetholone	Coronary artery	10000	None	Improved	^[9]^
Plasminogen Tochigi	Cerebral artery, splenic artery, renal artery	3000	Warfarin	Improved	^[10]^
Antiphospholipid syndrome	Cerebral venous sinus	22000	Aspirin, mephylprednisolone, CSP	Improved	^[11]^
Danazol	Cerebral venous	90000	Low molecular weight heparin	Improved	^[12]^
Platelet transfusion	Coronary stent	12000	Balloon angioplasty	Improved	^[13]^
PNH clone	Superior mesenteric vein, portal vein	29000	Warfarin	Improved	^[14]^
CSP	Cerebral venous sinus thrombosis	55000	Enoxaparin	Improved	^[15]^
Eltrombopag	Venous thrombus	NA	NA	NA	^[16]^
Eltrombopag, PNH clone	Left atrial appendage, ischemic colitis	13000	Supportive	Improved	Present case

The presumed causes in the literature were anabolic hormone administration in three cases [9,12], congenital coagulation gene abnormality in one case [9], antiphospholipid antibody syndrome in one case [11], platelet transfusion in one case [13], PNH blood cell involvement in one case [13], and CSP in one case [15]. We believe that eltrombopag was one of the causes in this case, but the presence of PNH blood cells and the fact that the patient was receiving CSP and platelet transfusion may also have been involved in the thrombus development. In the present case, atrial fibrillation induced a thrombus in the left ear, which may have led to ischemic enterocolitis, even though the patient's PT-INR was well within therapeutic range with warfarin and rather prolonged due to the interaction of CSP. Although observation of the colonic mucosa by endoscopy is essential for diagnosing hematogenous enteritis, colonoscopy was not performed in this case because of thrombocytopenia. The patient had comorbidities such as atrial fibrillation and constipation prior to the onset of aplastic anemia, which may have been associated with the development of this thrombotic event. It is possible that the decreased blood flow to the colonic mucosa associated with increased abdominal pressure during defecation was a contributing factor to ischemic enteritis. It was unlikely that malignancy caused the thrombosis since no obvious malignant complications were found on CT scan examination or other screening. Since there was no activated partial thromboplastin time (APTT) prolongation, we considered it unlikely that antiphospholipid antibody syndrome was the cause. As for thrombus formation, eltrombopag may have increased p-selectin and other substances on the platelet surface, and platelet transfusion and other factors may have also contributed to platelet aggregation. Alternately, PNH blood cells, such as platelets, monocytes, and neutrophils without the complement regulator CD59, promote thrombus formation and may cause thrombotic events in this case [17,18]. Regarding the platelet count at the time of thrombosis, in all previous reports, including the present case, thrombosis occurred with a platelet count lower than the normal range. However, some cases occurred after platelet transfusion or during platelet count recovery. The patient was treated for aplastic anemia with G-CSF, which has been reported to act directly to aggregate platelets [19]. It is unclear to what extent G-CSF affects thrombosis when platelet counts are low, but the combination of factors, TPO-RA, G-CSF, steroid administration, and PNH blood cells might act synergistically on thrombogenesis. After the thrombotic event had calmed down, eltrombopag was resumed at a low dose of 25 mg, and the dose was not increased for a long time. After that, the thrombosis did not recur. The low dose of eltrombopag did not cause the recurrence of thrombosis, and the absence of other concomitant medications, such as G-CSF or steroids, did not cause thrombosis.

## Conclusions

TPO-RAs, such as eltrombopag and romiplostim, are now widely used for AA. We must remember that thrombotic complications can occur during platelet recovery and when platelet counts are low. When administering these agents, it is essential to monitor platelet counts and the coagulation system regularly, with attention to atrial fibrillation and other thromboembolic risk factors, and to decide whether to continue or discontinue anticoagulation therapy. The treatment of patients with AA who develop thromboembolism and pancytopenia after TPO-RA therapy can be challenging; however, with appropriate supportive care, thromboembolism and AA can be controlled.
